# Phase I Hydroxylated Metabolites of the K2 Synthetic Cannabinoid JWH-018 Retain *In Vitro* and *In Vivo* Cannabinoid 1 Receptor Affinity and Activity

**DOI:** 10.1371/journal.pone.0021917

**Published:** 2011-07-06

**Authors:** Lisa K. Brents, Emily E. Reichard, Sarah M. Zimmerman, Jeffery H. Moran, William E. Fantegrossi, Paul L. Prather

**Affiliations:** 1 Department of Pharmacology and Toxicology, University of Arkansas for Medical Sciences, Little Rock, Arkansas, United States of America; 2 Public Health Laboratory, Arkansas Department of Health, Little Rock, Arkansas, United States of America; The Scripps Research Institute, United States of America

## Abstract

**Background:**

K2 products are synthetic cannabinoid-laced, marijuana-like drugs of abuse, use of which is often associated with clinical symptoms atypical of marijuana use, including hypertension, agitation, hallucinations, psychosis, seizures and panic attacks. JWH-018, a prevalent K2 synthetic cannabinoid, is structurally distinct from Δ^9^-THC, the main psychoactive ingredient in marijuana. Since even subtle structural differences can lead to differential metabolism, formation of novel, biologically active metabolites may be responsible for the distinct effects associated with K2 use. The present study proposes that K2's high adverse effect occurrence is due, at least in part, to distinct JWH-018 metabolite activity at the cannabinoid 1 receptor (CB1R).

**Methods/Principal Findings:**

JWH-018, five potential monohydroxylated metabolites (M1–M5), and one carboxy metabolite (M6) were examined in mouse brain homogenates containing CB1Rs, first for CB1R affinity using a competition binding assay employing the cannabinoid receptor radioligand [^3^H]CP-55,940, and then for CB1R intrinsic efficacy using an [^35^S]GTPγS binding assay. JWH-018 and M1–M5 bound CB1Rs with high affinity, exhibiting K_i_ values that were lower than or equivalent to Δ^9^-THC. These molecules also stimulated G-proteins with equal or greater efficacy relative to Δ^9^-THC, a CB1R partial agonist. Most importantly, JWH-018, M2, M3, and M5 produced full CB1R agonist levels of activation. CB1R-mediated activation was demonstrated by blockade with O-2050, a CB1R-selective neutral antagonist. Similar to Δ^9^-THC, JWH-018 and M1 produced a marked depression of locomotor activity and core body temperature in mice that were both blocked by the CB1R-preferring antagonist/inverse agonist AM251.

**Conclusions/Significance:**

Unlike metabolites of most drugs, the studied JWH-018 monohydroxylated compounds, but not the carboxy metabolite, retain *in vitro* and *in vivo* activity at CB1Rs. These observations, combined with higher CB1R affinity and activity relative to Δ^9^-THC, may contribute to the greater prevalence of adverse effects observed with JWH-018-containing products relative to cannabis.

## Introduction

In recent years, several products sold as incense in headshops, commonly referred to as “K2” and “Spice”, have rapidly emerged as legal substitutes for cannabis due to their cannabimimetic effects when smoked or consumed [1]. Though marketed as “natural” herbal blends, K2 products are usually non-psychotropic plant matter adulterated with various synthetic cannabinoids, most of which are aminoalkylindoles (AAIs) of the JWH family, a series of WIN-55,212-2 analogues created in 1994 by Dr. John W. Huffman for structure-activity relationship studies of the cannabinoid receptors [2], [3]. They, along with other synthetic cannabinoids, such as CP-47,497 and HU-210, were first found in the “natural” herbal blends in 2008 [1], [4], [5], [6]. One particular AAI, JWH-018 [7] is quite prevalent across many different brands and batches of K2 products [8], [9]. JWH-018 and other cannabinoids, such as Δ^9^-tetrahydrocannabinol (Δ^9^-THC), the major active constituent in marijuana, produce their psychoactivity by binding and activating, to varying degrees, cannabinoid 1 receptors (CB1Rs) in the CNS, which are G_i/o_-protein coupled receptors (GPCRs) [10]. Although the desired effects of K2 products are generally similar to those of marijuana, the adverse effect frequency and severity of K2 is much greater than that of marijuana, which has been used for millennia and is the most commonly abused illegal drug in the U.S. [11]. While smoking or oral consumption of marijuana acutely produces relatively mild and tolerable side effects in most users, such as appetite stimulation and orthostatic hypotension, it very rarely causes the adverse effects observed rather commonly with similar use of K2 products, such as hypertension, agitation, hallucinations, psychoses, seizures and panic attacks [1], [4], [12], [13]. In extreme THC overdose cases, similar symptoms can be observed but they are not typically associated with THC use. In addition to acute adverse effects produced by K2, a case report indicates that chronic abuse may also result in a severe withdrawal and dependence syndrome [14]. The use of K2 has even been causally linked to at least one death by overdose and has been implicated for likely involvement in several other fatalities, resulting in over 2500 calls to poison control centers in 2010 alone and numerous visits to emergency departments across the United States [15], [16], [17] and Europe [13], [18].

These observations have garnered the attention of public health and legislative officials in many municipalities, and have even moved the US Drug Enforcement Administration (DEA) to use its emergency powers to temporarily categorize JWH-018 and four other synthetic cannabinoids as Schedule I substances for at least one year because “…they impose imminent hazard to public safety” [19]. Regardless of proactive legislative movements, these products are still legal and available in most countries throughout the world. Furthermore, the rapid increased use of K2 products among youth, their current inability to be detected by standard drug urine tests and the constant introduction of new structurally similar products of unknown content pose a significant risk to public health. Most importantly, the pharmacological and toxicological profiles of these products are virtually unknown, and the mechanisms underlying the discrepancies in the adverse effect frequency and severity of K2 relative to the well-established cannabis have yet to be elucidated.

JWH-018 activates CB1Rs with greater potency and efficacy than Δ^9^-THC [10], [20]. While such pharmacodynamic differences may explain some reports of toxicity, such as overdosing by users expecting a cannabis-like potency, other mechanisms may also be responsible for the distinct discrepancy in side effect severity often observed for these two CB1R agonists. For instance, even very high doses of marijuana are unlikely to acutely cause seizures or permanent cardiac damage in otherwise healthy individuals as it has been reported for K2 [21]. One likely mechanism underlying these observations might result from unique pharmacokinetic profiles for these two structurally distinct cannabinoids. For example, although both compounds undergo Phase I metabolism by cytochromes P450, JWH-018 has been shown to have at least nine monohydroxylated metabolites whose biological activity is currently undetermined [9], while Δ^9^-THC has only one known major psychoactive monohydroxylated metabolite, 11-OH-THC [22]. Both Δ^9^-THC and JWH-018 have been shown to be metabolized to one primary carboxy metabolite [22], [23]. Mass spectral analysis of urine and serum samples collected from K2 users have also shown appreciable concentrations of several of the hydroxylated JWH-018 metabolites [8], [23] and our recent study using authentic standards [24] confirmed that humans excrete four of the metabolites examined in the current study (M2, M3, M5, and M6) at levels ranging from 12 to 83 ng/mL. Although Phase I hydroxylation is generally considered to result in an inactivation of parent compounds, it is nevertheless possible that one or more JWH-018 metabolites might instead display distinct pharmacological and/or toxicological properties. Therefore, we hypothesized that the discrepancies in adverse effect frequency and severity reported for Δ^9^-THC relative to K2 might be due, in part, to differences in the action of distinct monohydroxylated or carboxy metabolites of JWH-018 relative to Δ^9^-THC.

**Figure 1:**
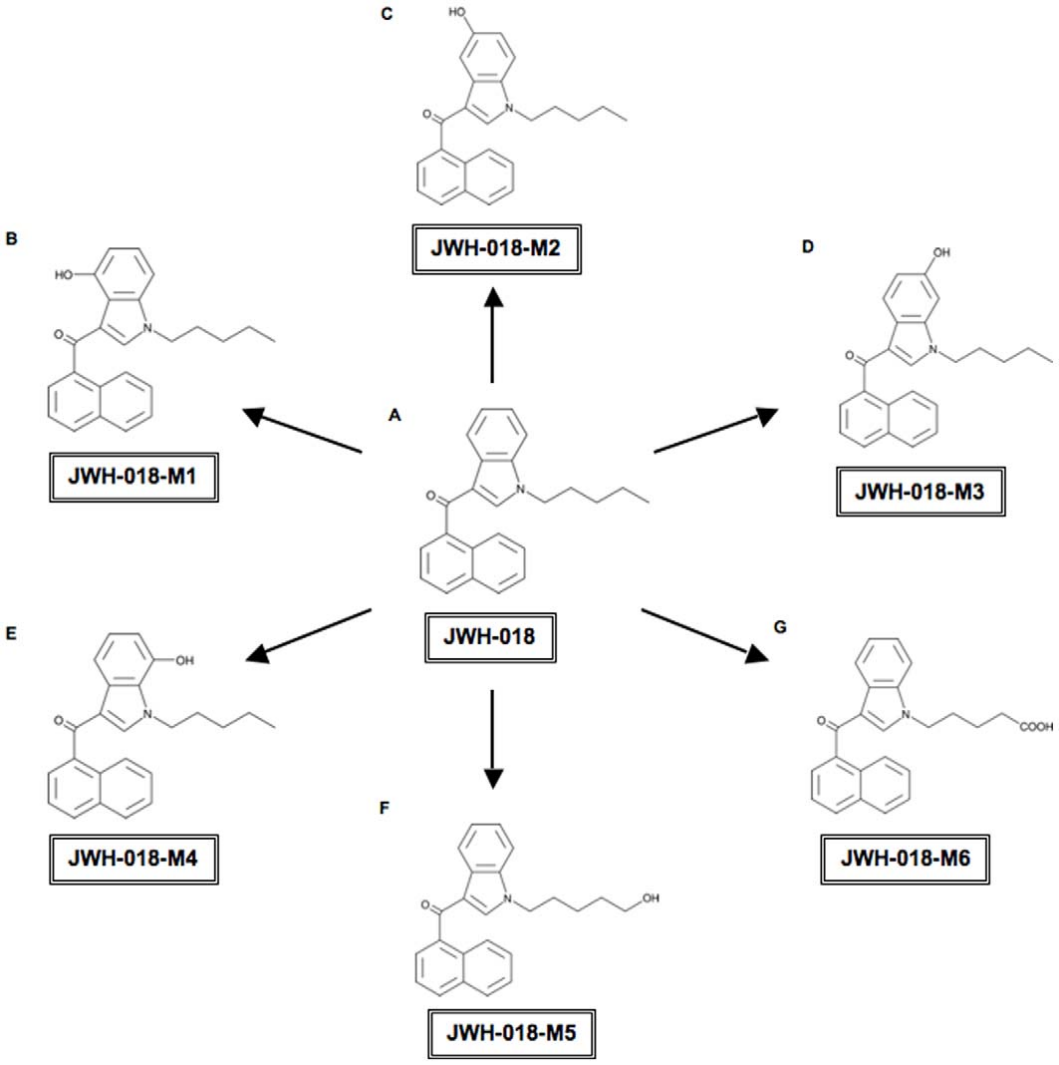
Structures of JWH-018 and six JWH-018 hydroxylated products. **A. JWH-018** [(1-pentyl-1H-indol-3-yl)-1-naphthalenyl-methanone] **B. M1** [(4-hydroxy-1-pentyl-1H-indol-3-yl)(naphthalen-1-yl)methanone] **C. M2** [(5-hydroxy-1-pentyl-1H-indol-3-yl)(naphthalen-1-yl)methanone] **D. M3** [(6-hydroxy-1-pentyl-1H-indol-3-yl)(naphthalen-1-yl)methanone] **E. M4** [(7-hydroxy-1-pentyl-1H-indol-3-yl)naphthalen-1-yl)methanone] **F. M5** [(1-(5-hydroxypentyl)-1H-indol-3-yl)(naphthalen-1-yl)methanone] **G. M6** [5-(3-(1-naphthoyl)-1H-indol-1-yl)pentanoic acid].

This hypothesis was tested in the present study by determining the *in vitro* affinity for and intrinsic activity at CB1Rs of five monohydroxylated (M1–M5, Fig. 1) and one carboxy derivative of JWH-018 (M6, Fig. 1). Furthermore, to establish *in vivo* relevance, the activity of JWH-018 and one of its metabolites exhibiting similar *in vitro* potency and efficacy to Δ^9^-THC were evaluated in NIH Swiss mice by examining two endpoints from the standard cannabinoid tetrad battery of tests. We report that several hydroxylated derivatives of JWH-018 not only retain nanomolar binding affinity for CB1Rs, but also exhibit a range of intrinsic activities from partial to full agonism. This new information is critical for understanding the pharmacological significance of JWH-018 metabolites produced in humans.

## Results

### Five JWH-018 hydroxylated metabolites bind mouse CB1R with affinities greater than or equal to that of Δ^9^-THC

**Figure 2:**
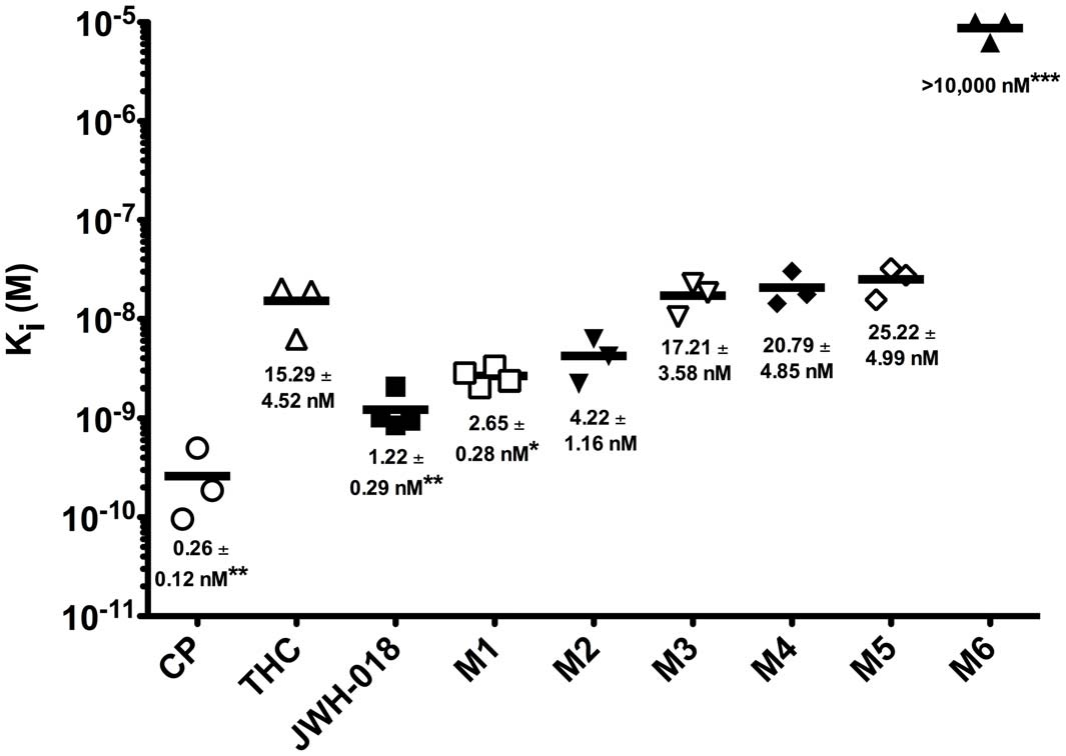
JWH-018 and M1–M5 bind CB1R with equal or greater affinity than Δ^9^-THC. JWH-018 and M1, M2, M3, M4, M5, but not M6, completely displaced the radiolabeled cannabinoid [^3^H]CP-55,940 from CB1Rs (data not shown). Affinities for CB1Rs of JWH-018 and M1–M5 were equivalent to or up to 10-fold greater than that of Δ^9^-THC (*P<0.05, **P<0.01, ***P<0.001 relative to Δ^9^-THC, one way ANOVA with Dunnett's Multiple Comparison Test, n = 3–4).

The cannabis-like actions of JWH-018-containing products in human users indicate a CB1R-dependent mechanism of action for the parent compound and, potentially, its metabolites. Although JWH-018 has been shown to bind to CB1Rs with high affinity [10], to date, the action of its metabolites at this receptor is unknown. Therefore, the present study employed a radiolabeled competition binding assay to determine the affinity (K_i_) of JWH-018 and five monohydroxylated derivatives (M1–M5, Fig. 1) and one carboxy metabolite (M6, Fig. 1) to CB1Rs in mouse whole brain membrane homogenates. CB1Rs are endogenously expressed in abundant quantities in the CNS, while negligible levels of CB2 receptors are present [25], [26]. Therefore, brain tissue provides a concentrated source of CB1Rs that is practically devoid of CB2Rs and is therefore generally accepted for use in *in vitro* CB1R studies [27], [28]. Cannabinoids and all tested JWH-018 compounds (M1–M6) were evaluated for the ability of increasing concentrations to compete against the radiolabeled cannabinoid [^3^H]CP-55,940 for binding to CB1Rs. Data are expressed as the percent specific binding occurring at each drug concentration relative to the level of binding present in the presence of vehicle only. Saturation binding with [^3^H]CP-55,940 showed that mouse brain homogenates contain a CB1R density of 2.44±0.15 pmole/mg protein, to which [^3^H]CP-55,940 binds with an affinity (K_d_) of 0.37±0.07 nM (n = 3, data not shown). Δ^9^-THC, JWH-018 and M1–M5 produce 100% displacement of [^3^H]CP-55,940 from CB1Rs (data not shown) and bind with affinities (K_i_) in the low 2–30 nM range with a rank order of JWH-018 = M1>M2>Δ^9^-THC = M3 = M4 = M5≫M6 (Fig. 2). Importantly, M1 retains CB1R affinity (2.6±0.3 nM) similar to that of the parent compound (1.2±0.3 nM), while the carboxy derivative M6 fails to bind to CB1Rs. It is also significant to note that JWH-018 and M1 bind to CB1Rs with almost 10-fold higher affinity than Δ^9^-THC (15.29±4.5 nM) and all other tested compounds (except M6) also bind to mCB1Rs with equivalent affinity relative to Δ^9^-THC. The Ki values determined here for CP-55,940, Δ^9^- THC, and JWH-018 agree well with those previously reported for these cannabinoids of 0.5–5 nM, 5.05–80.3 nM, and 9.0 nM, respectively [29], [30].

### JWH-018 and M1–M5 activate mouse CB1Rs with a range of low partial to full agonism

**Figure 3:**
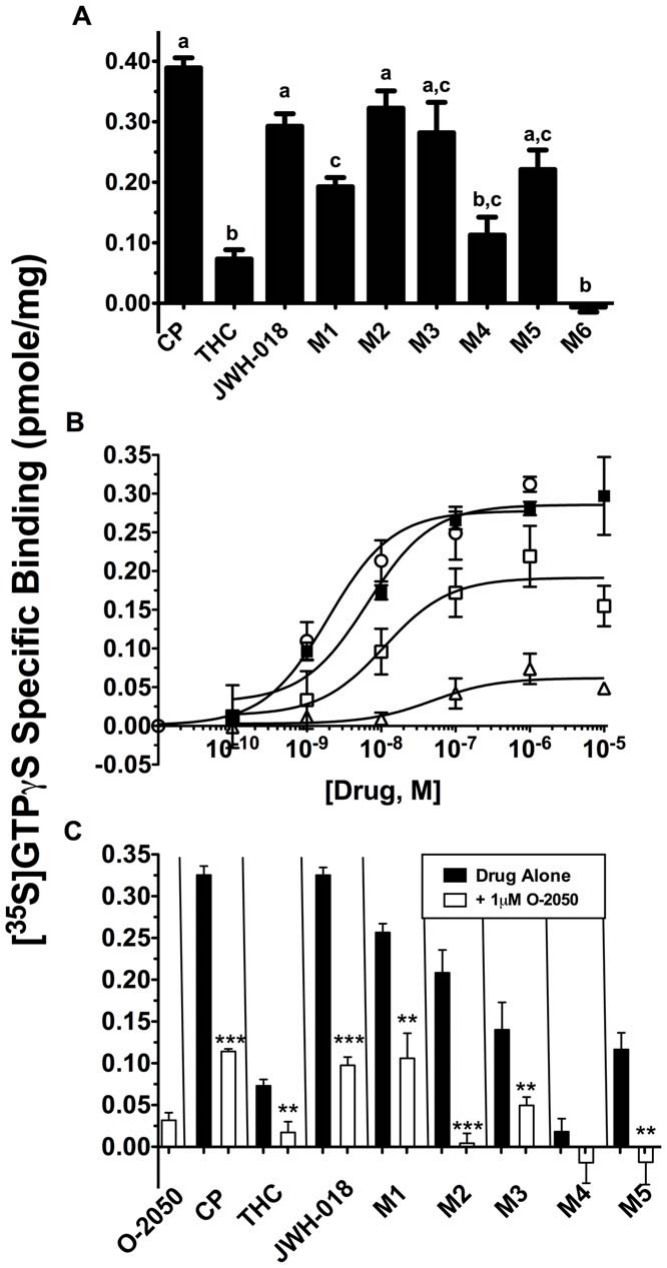
JWH-018 and M1–M5 activate CB1R. **A.** Ten µM concentrations of JWH-018, M1, M2, M3, and M5 activated brain GPCRs greater than 10 µM Δ^9^-THC. Activation by JWH-018, M2, M3 and M5 did not differ from the full CB1R agonist CP-55,940. Values designated with different letters above the error bars are significantly different (P<0.05, one way ANOVA with Tukey's Multiple Comparison *post-hoc* Test, n = 3–10). **B.** JWH-018 and M1 stimulated G-proteins more potently and efficaciously than Δ^9^-THC, n = 3–4. **C.** GPCR activation by an estimated ED_90_ concentration (100 nM) of metabolites was blocked by co-incubation with 1 µM of the selective neutral CB1R antagonist O-2050 (**P<0.01, ***P<0.001 vs drug alone, Student's *t*-test, n = 3–7).

Subsequent studies employing the [^35^S]GTPγS binding assay characterized the intrinsic efficacy of these compounds at CB1Rs by examining their ability to activate G-proteins (Fig. 3). Similar to previous reports [31], a receptor saturating concentration (10 µM) of the full CB1R agonist CP-55,940 produces over 5-fold greater G-protein stimulation than a similar maximal concentration of the partial CB1R agonist Δ^9^-THC (0.39±0.02 vs. 0.07±0.01 pmole/mg, Fig. 3A). JWH-018 and M1–M5 act as partial (Δ^9^-THC, M1, M4) or full (CP-55,940, JWH-018, M2, M3, M5) agonists at mCB1Rs. For example, a receptor saturating concentration of JWH-018 and M2 produces 0.29±0.02 and 0.32±0.03 pmole/mg stimulation of G-proteins, respectively. Although this level of activation is slightly less, it is not significantly different than the amount of activation produced by the full agonist CP-55,940. Importantly, JWH-018 and 5 of the 6 oxidized products of JWH-018 tested produce equivalent (M4) or greater (JWH-018, M1, M2, M3, M5) levels of G-protein stimulation than Δ^9^-THC. The activation of G-proteins produced by CP-55,940, JWH-018, M1 and Δ^9^-THC is also concentration-dependent (Fig. 3B) and occurs with an identical rank order of potency as predicted by the affinity of these compounds for CB1Rs, with EC_50_ values of 3.36±2.35 nM, 6.82±2.48, 17.01±9.59 and 167.4±84.7 nM, respectively (Fig. 2). In agreement to data presented in Fig. 3A, the maximal efficacy (E_max_) of G-protein activation in this assay for CP-55,940, JWH-018, M1 and Δ^9^-THC were 0.28±0.02 pmole/mg, 0.29±0.02 pmole/mg, 0.19±0.02, and 0.06±0.01. Concentration-dependence and agreement between the rank order of receptor affinity and the potency for G-protein activation provide strong evidence for a receptor-mediated mechanism, most likely via CB1Rs. Lastly, G-protein activation produced by an ED_90_ concentration of all cannabinoids and metabolites examined (*e.g.*, 100 nM, estimated from Fig. 3B) was significantly attenuated by co-incubation with a receptor saturating concentration (1 µM) of the selective CB1R neutral antagonist O-2050 (Fig. 3C).

### Δ^9^-THC, JWH-018 and M1 reduce locomotor activity and core body temperature by a CB1R-dependent mechanism

**Figure 4:**
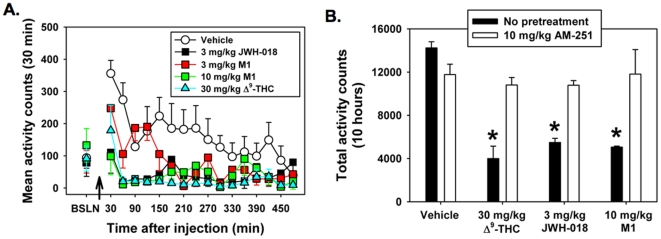
JWH-018 and M1 decreased mouse locomotor activity in a CB1R-dependent manner, similar to Δ^9^-THC. **A.** Intraperitoneal (i.p.) administration of 3 mg/kg JWH-018, 10 mg/kg JWH-018 M1, and 30 mg/kg Δ^9^-THC decreased locomotor activity relative to vehicle controls over a 10 h time course, beginning 60 min after injection. **B.** Area under the curve data generated from the 10 h time-course shows 3 mg/kg JWH-018, 10 mg/kg JWH-018 M1, and 30 mg/kg Δ^9^-THC significantly decrease locomotor activity relative to vehicle controls (*P<0.05 vs. vehicle controls, Kruskal-Wallis one-way ANOVA with Tukey HSD test, n = 5). Co-administration of each cannabinoid with the CB1R-preferring antagonist/inverse agonist AM251 (10 mg/kg) restored locomotor activity to vehicle control levels.

**Figure 5:**
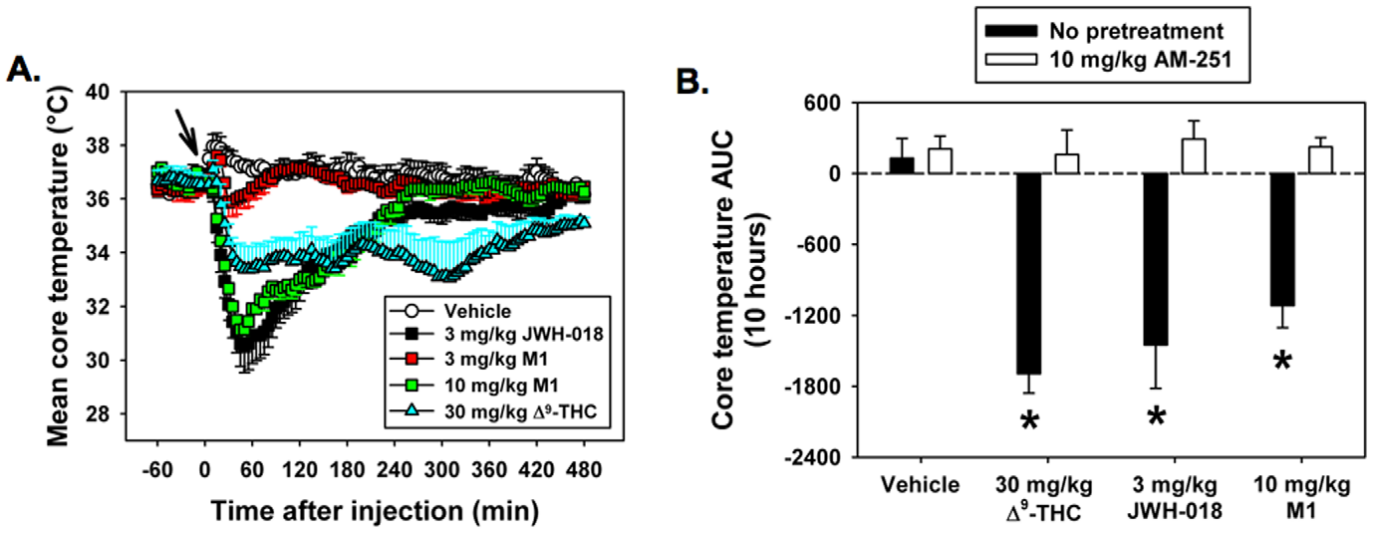
JWH-018 and M1 decreased mouse core body temperature in a CB1R-dependent manner similar to Δ^9^-THC. **A.** Mice administered 3 mg/kg JWH-018 and 10 mg/kg M1 (i.p.) exhibited greater depressions in core body temperature than 30 mg/kg Δ^9^-THC, but also recovered more quickly over a 10 h time course, resulting in **B.** equivalent area under the curve values, which were significantly lower than vehicle controls (*P<0.005 vs. vehicle controls, one-way ANOVA with Tukey HSD test, n = 5). Core body temperature was restored to vehicle control levels by coadministration of cannabinoids with the CB1R-preferring antagonist/inverse agonist AM251 (10 mg/kg).

The specific mechanism underlying whole-animal effects produced by suspected cannabinoids can be validated *in vivo* by measuring standard, well-documented physiological parameters that are consistently altered by CB1R activation in rodents (*e.g.*, the cannabinoid “tetrad” [32]). In the present study, two of the four parameters associated with the cannabinoid tetrad, locomotor activity and core body temperature, were measured in mice. As anticipated, both physiological parameters are sharply depressed by intraperitoneal (i.p.) administration of 30 mg/kg Δ^9^-THC (Figs. 4 and 5). Furthermore, i.p. administration of 3 mg/kg of JWH-018 and 10 mg/kg M1 also reduces natural exploratory behavior in a novel environment to levels equivalent to that produced by 30 mg/kg of Δ^9^-THC over a 10 hr observation period (Fig. 4A). The effect of Δ^9^-THC, JWH-018 and M1 on locomotor activity is significantly attenuated by pretreatment of mice with a 10 mg/kg dose of the CB1-preferring antagonist/inverse agonist AM-251 (Fig. 4B) (*P*≤0.001 across all groups, H = 30.000 with 7 DF). The same doses of all three compounds also produce a significant decrease in core body temperature relative to vehicle-treated controls, beginning between 30 and 60 min after injection (P<0.001 across all groups, F = 15.704 with 7 DF). Peak temperature depression, as well as the rate of temperature recovery is much greater for JWH-018 and M1 than for Δ^9^-THC (Fig. 5A) and is ultimately reflected by similar area under the curve values when averaged across the entire 10 hr observation period (Fig. 5B). Similar to effects on locomotor activity, pretreatment with AM-251 (10 mg/kg) restored core body temperature to control levels, signifying a CB1R-dependent mechanism.

## Discussion

This study importantly demonstrates for the first time that five potential Phase I hydroxylated metabolites of the synthetic cannabinoid JWH-018 bind with high nanomolar affinity to, and very efficaciously activate, CB1Rs *in vitro*. Furthermore, the sharp decrease in locomotor activity and core body temperature in mice produced by the M1 derivative of JWH-018 and the reversal of these effects by a CB1R-preferring antagonist indicate that potential metabolites of this emerging drug of abuse are active *in vivo* as well. Importantly, JWH-018 and most of the tested derivatives apparently elicit greater *in vitro* and *in vivo* responses relative to Δ^9^-THC, the well-known classical cannabinoid present in marijuana. By comparison, all Δ^9^-THC metabolites, except one, are inactivated by oxidative metabolism, which prevents further CB1R activation. The higher affinity, potency and efficacy of JWH-018, coupled with its potential metabolism to a number of equally active metabolites, suggests that both acute and chronic effects of JWH-018 might be intensified when compared to a similar level of exposure to Δ^9^-THC. Taken collectively, the results presented here suggest that differences in both the pharmacodynamic and pharmacokinetic properties of JWH-018 relative to Δ^9^-THC might help to explain the distinct adverse clinical manifestations often observed with K2 use. While the present study reveals the ability of these JWH-018 derivatives to act at CB1Rs, pharmacokinetic analysis will be required to definitively determine the presence of these metabolites in target tissues in collective concentrations high enough to elicit adverse effects *in vivo*. The recent discovery of detectable nanomolar concentrations of M2, M3, and M5, the most efficacious of the JWH-018 derivatives examined in this study, in human urine [24], supports the current proposal that metabolites contribute to the effects of K2 and should thus be pursued further.

The emergence of “legal highs” in response to synthetic cannabinoid use is relatively new [5] and the field is still in its infancy; hence, limited studies have examined the pharmacological or toxicological properties of K2 products and their active components. Moreover, the main foci of the current K2 literature are only clinical case studies reported from emergency departments [12], [13], [14], [15], [16], [18] and methods detailing the analytical detection of synthetic K2 cannabinoids in body fluids [4], [6], [8], [23], [24], [33]. For these reasons, coupled with the rapidly growing use and dangerous adverse effect profile of K2 products, it is critical that clinicians and basic scientists obtain a strong mechanistic understanding of the cannabinoid constituents in K2, so these drugs can be identified, regulated and therapies designed to address the adverse effects.

Currently, little is known regarding the structure-activity relationships (SARs) at CB1R of aminoalkyindole cannabimimetics that are substituted around the indole nucleus, especially at positions 4–7. Although Eissenstat et al. [34] examined a number of substitutions, their significant findings focused on the importance of substitutions at positions 1–3. To our knowledge, the present work is the first to show that hydroxylation at positions 5 and 6 (metabolites M2 and M3) retain significantly higher activity, with little difference in affinity, compared to hydroxylation at positions 4 and 7 (M1 and M4). The importance of the substituent at the end of the pentyl chain is also underscored in the present work by the observation that addition of a carboxylic acid (M6), but not a hydroxyl group (M5) totally eliminates affinity for CB1R.

Detailed characterization of the specific enzymes responsible for biotransformation of JWH-018 and structurally similar synthetic cannabinoids would be an important step to fully understand the consequences of JWH-018 use in humans. A recent report, employing crude human liver microsomes, suggests that specific isoforms of the cytochromes P450 system are essential for metabolism of JWH-018 to various mono- and dihydroxylated metabolites [9], including the metabolites examined in the present study. Likewise, a second *in vitro* study, using rat liver microsomes [35], has identified the cytochrome P450 system responsible for metabolism of the structurally similar CB2 selective agonist JWH-015. However, to our knowledge, no study has reported the *specific* enzymes responsible for production of the primary and secondary metabolites of JWH-018. When delineated, SARs, mutagenesis and polymorphism studies of these specific enzymes might reveal inter-individual differences for production of active metabolites and thus provide support for why some individuals exhibit greater degrees of severe adverse reactions to K2 exposure. If correlated to clinical observations, this information could provide a model to predict severe adverse effects in susceptible individuals. For example, different enzymatic polymorphisms may yield distinct metabolic rates that vary from person to person and produce a preference for formation of certain metabolites relative to others. As predicted by the present study, because different metabolites exhibit various degrees of activity, a bias towards production of more active metabolites that could increase the net activation of CB1R is possible. Alternatively, it might be predicted that production of less active metabolites by certain individuals would result in antagonistic effects with concurrently administered cannabinoids, potentially leading to greater use of synthetic cannabinoids in an attempt to overcome the reduction in effects. Although speculative, it is possible that biased metabolic profiles could produce a mix of active metabolites, producing a multitude of “entourage effects” associated with use of JWH-018. Such complex effects could have unique and potentially harmful consequences on the delicate balance of the endocannabinoid system, which plays important roles in modulating mood [36], appetite and energy homeostasis [37], [38], pain sensation [39], immune function [40], fertility [41] and possibly bone homeostasis [42].

The [^35^S]GTPγS binding experiments presented in the current study indicate that several potential JWH-018 hydroxylated metabolites activate G-proteins in mouse brain that cannot be completely antagonized by the CB1R neutral antagonist O-2050. Although several explanations are possible, cannabinoid receptor-independent GPCR activation in response to K2 use is probable. The possibility of CB1R-independence, both GPCR and non-GPCR mediated, is supported by the clinical observations of seizures, hallucinations, anxiety, agitation, panic attacks, and hypertension, which are not typically observed following CB1R activation. The mechanisms behind these atypical adverse effects, although not completely understood themselves, nevertheless give additional information as to how K2 is acting *in vivo*. For example, grand mal seizures, which can occur with K2 use, are the result of excessive, aberrant neural synaptic firing that leads to involuntary tonic-clonic spasms. The mechanisms behind grand mal seizures are complex and diverse, but ultimately involve disinhibition of excitatory neurons [43]. Retrograde activation of CB1Rs by cannabinoids and endocannabinoids hyperpolarizes presynaptic neurons and thus inhibits synaptic transmission [44], and several cannabinoids have even been shown to exhibit anticonvulsive activity [45], [46], [47], [48]. Therefore, seizures caused by K2 are possibly due to the antagonism of other inhibitory networks, such as GABA channels [49], and/or the activation of excitatory networks, such as metabotropic glutamate receptors (mGluRs) [50], Na^+^ channels [51], and Ca^2+^ channels [43]. Hallucinations, as well as psychosis in susceptible individuals with a previous personal and/or family psychiatric history [12], [13], have also been associated with K2 use. Theories underlying the neurobiological mechanisms of hallucinations and psychosis include abnormal dopaminergeric neurotransmission, as described in the dopamine hypothesis of schizophrenia [52], serotonergic transmission, as seen with the serotonergic classical hallucinogens [53], and NMDA glutamate receptor blockade [54]. The cardiovascular symptoms, as well as drug-induced anxiety, agitation and panic attacks, associated with K2 use could be caused by activation of α_1_, β_1_ and β_2_ adrenoceptors [55], [56]. Activation of mGluRs [50], as well as GABA channel blockade [57], may also be responsible for anxiety due to K2. Alteration of the receptor networks mentioned here are just a few examples of many possible that may result in severe adverse effects seen in an alarmingly large proportion of K2 users.

The present study investigates some previously unknown actions of oxidized products of JWH-018 produced by using the relatively new and increasingly common drug of abuse, K2. Although JWH-018 is a predominant component of K2, it is unfortunately only one of a whole host of cannabimimetic compounds found in varying, unregulated concentrations from brand-to-brand and, even within brands, batch-to-batch of K2. This reality presents a challenge to researchers and clinicians in their attempts to better understand and predict the biological consequences of K2 use and thus accurately warn the general public about its risks, as well as advise legislators, who are currently working to determine the appropriate legal status of K2. The uncontrolled and heterogeneous nature of K2 also presents a danger to even its more experienced users who may unknowingly use K2 containing particular synthetic cannabinoid blends to which they may have an adverse reaction. Nonetheless, this work represents an important initial step toward understanding K2 by uncovering significant CB1R affinities and intrinsic activities of five potential metabolites of JWH-018. Many of the synthetic cannabinoids found in K2 are aminoalkylindoles of the JWH family and are quite structurally similar to JWH-018. Since JWH-018 produces metabolites with partial to full agonist activity at CB1Rs, it is justified to posit that similar K2 synthetic cannabinoids can also be biotransformed into molecules with various levels of affinity and activity at CB1Rs, as well as at other receptor systems as discussed above. Altogether, the presence of parent synthetic cannabinoid molecules within a single dose of K2, combined with the respective active metabolites produced, could conceivably act in concert to produce the dynamic range of effects observed following use of various K2 preparations. The idea that active metabolites are generated from not just one, but several parent molecules found within a single drug of abuse, is novel and exciting, but complicates matters by introducing an intrinsic polypharmacy effect. Therefore, much future investigation is required to fully elucidate human metabolic products to better assess which oxidized products of JWH-018 retain pharmacological activity, the relative contributions of each product and any synergistic/antagonistic interactions between molecules. In conclusion, the discovery that JWH-018 metabolites, and other oxidized products of JWH-018, partake actively and diversely in the activity of K2 provides a substantial avenue of exploration and thus serves as an essential building block in combating problems associated with an increasingly common drug of abuse.

## Materials and Methods

### Materials

All drugs used for *in vitro* assays were diluted to a stock concentration of 10^−3^ M with 100% ethanol and stored at −20°C. JWH-018 and its potential metabolites (M1–M6) were purchased from Cayman Chemical (Ann Arbor, MI), who chemically synthesized the metabolites and determined structures through mass spectrometry and NMR. Δ^9^-THC was supplied by the National Institute on Drug Abuse (NIDA, Bethesda, MD). WIN-55, 212-2, CP-55,940, AM-251, and O-2050 were purchased from Tocris Bioscience (Ellisville, MO). GTPγS and GDP used in the [^35^S]GTPγS assay were purchased from EMD Chemical (Gibbstown, NJ), and Sigma Aldrich (St. Louis, MO), respectively. Both chemicals were diluted to a stock concentration of 10^−2^ M with water and stored at −20°C. [^3^H]CP-55,940 (174.6 Ci/mmol) used for competition receptor binding was purchased from PerkinElmer (Waltham, MA) and [^35^S]GTPγS (1250 Ci/mmol) was purchased from American Radiolabeled Chemicals (St. Louis, MO). For *in vivo* studies, all drugs were dissolved to the appropriate concentrations in a ratio of 1∶1∶18 of absolute ethanol∶ emulphor∶ physiological saline vehicle and stored at 4°C until used.

### Membrane Preparation

Whole brains were harvested from decapitated B6SJL mice, snap-frozen in liquid nitrogen, and stored at −80°C. To prepare crude membrane homogenates, brains were thawed on ice, pooled and suspended in ice-cold homogenization buffer (50 mM HEPES pH 7.4, 3 mM MgCl_2_, and 1 mM EGTA) [58]. Suspended brains were then subjected to 10 complete strokes employing a 40 mL Dounce glass homogenizer, and centrifuged at 40,000× *g* for 10 min at 4°C. Supernatants were discarded and pellets were resuspended in ice cold homogenization buffer, homogenized and centrifuged similarly twice more. Following the final centrifugation step, pellets were resuspended in ice-cold 50 mM HEPES, pH 7.4, to a concentration of approximately 2 mg/mL and aliquoted for storage at −80°C. Protein concentration was determined using BCA™ Protein Assay (Thermo Scientific, Rockford, IL).

### Competition Receptor Binding Assay

Fifty µg of mouse brain membrane homogenates (containing a relatively pure source of CB1Rs) were incubated with 0.2 nM of the radiolabeled cannabinoid agonist [^3^H]CP-55,940 for 90 min at room temperature in an assay buffer containing 5 mM MgCl_2_, 50 mM Tris, 0.05% bovine serum albumin (BSA) and increasing concentrations (0.1 nM–10 µM) of JWH-018 M1–M6, or non-radioactive CP-55,940. Assays were performed in triplicate, in a final volume of 1 mL, as previously described [59]. Total binding was defined as the amount of radioactivity observed when 0.2 nM [^3^H]CP-55,940 was incubated in the absence of any competitor. Nonspecific binding was defined as the amount of [^3^H]CP-55,940 binding remaining in the presence of 10 µM of the non-radioactive CB1/CB2 cannabinoid agonist WIN-55,212-2. Specific binding was calculated by subtracting non-specific from total binding. Reactions were terminated by quick filtration through Whatman GF/B glass fiber filters, followed by five washes with an ice-cold buffer containing 50 mM Tris and 0.05% bovine serum albumin (BSA). Filters were punched out into 7 mL scintillation vials and immersed in 4 mL of ScintiVerse™ BD Cocktail scintillation fluid. After overnight extraction, bound radioactivity was determined by liquid scintillation spectrophotometry. Specific binding is expressed as a percentage of binding occurring in vehicle samples (*e.g.*, binding in the absence of any competitor).

### [^35^S]GTPγS Binding Assay

[^35^S]GTPγS binding was performed as previously described [60], with minor modifications. Each drug to be tested was incubated with 25 µg of mouse brain membrane homogenates, 10 µM GDP, 0.1 nM [^35^S]GTPγS and assay buffer (20 mM HEPES, 10 mM MgCl_2_, 100 mM NaCl, 20 units/L adenosine deaminase, 0.05% BSA). Assays were performed in triplicate in a final volume of 1 mL for 30 min at 30°C. Total binding was defined as the amount of radioactivity observed when 0.1 nM [^35^S]GTPγS was incubated in the absence of any cannabinoid. Nonspecific binding was defined as the amount of [^35^S]GTPγS binding remaining in the presence of 10 µM of non-radioactive GTPγS. Specific binding was calculated by subtracting non-specific from total binding. Reactions were terminated by quick filtration through Whatman GF/B glass fiber filters, followed by five washes with an ice-cold buffer containing 20 mM HEPES and 0.05% BSA. Filters were punched out into 7 mL scintillation vials and immersed in 4 mL of ScintiVerse™ BD Cocktail scintillation fluid. After overnight extraction, bound radioactivity was determined by liquid scintillation spectrophotometry. Specific binding is expressed as picomoles of [^35^S]GTPγS bound per mg of protein.

### Animal Care and Use

Prior to surgery (see below), male NIH Swiss mice (Harlan Sprague Dawley Inc., Indianapolis, IN), weighing approximately 25–30 g, were housed 3 animals per Plexiglas cage (15.24×25.40×12.70 cm) in a temperature-controlled room at the University of Arkansas for Medical Sciences. Room conditions were maintained at an ambient temperature of 22±2°C at 45–50% humidity. Lights were set to a 12-h light/dark cycle. Animals were fed Lab Diet rodent chow (Laboratory Rodent Diet #5001, PMI Feeds, Inc., St. Louis, MO) and water *ad libitum* until immediately before testing. Animals were acclimated to the laboratory environment 2 days prior to experiments and were tested in groups of 6 mice per condition. All studies were carried out in accordance with the Declaration of Helsinki and with the Guide for Care and Use of Laboratory animals as adopted and promulgated by the National Institutes of Health. Experimental protocols were approved by the Animal Care and Use Committee at the University of Arkansas for Medical Sciences (Animal Use Protocol #3155).

### Core Temperature and Locomotor Activity Measurements

Following appropriate anesthetization with ketamine (100 mg/kg, intraperitoneal [i.p.]) and xylazine (10 mg/kg, i.p.), the abdominal area of each mouse was shaved and sanitized with iodine swabs. A rostral-caudal cut approximately 1.5 cm in length was made with skin scissors, providing access to the intraperitoneal cavity. A cylindrical glass -encapsulated radiotelemetry probe (model ER-4000 E-Mitter, Mini Mitter, Bend, OR, USA) was then inserted, and the incision was closed using absorbable 5-0 chromic gut suture material. At least 7 days were imposed between surgery and experimental observation of drug effects to allow incisions to heal and mice to recover normal body weights. Following surgery, implanted mice were individually housed in Plexiglas mouse cages (15.24×25.40×12.70 cm) for the duration of all temperature and locomotor activity experiments. Implanted transmitters produced activity- and temperature-modulated signals that were transmitted to a receiver (model ER-4000 Receiver, Mini Mitter Co., Inc.) underneath each mouse cage. Receivers were housed in light- and sound-attenuating cubicles (Med Associates model ENV-022MD, St. Albans, VT) equipped with exhaust fans, which further masked ambient laboratory noise. On experimental days, mice were weighed, marked, and returned to their individual cages during which at least 1 hr of baseline data were collected. Cannabinoid doses were then calculated and drugs prepared for injection. Animals were subsequently removed from their cage and injected with various doses of Δ^9^-THC, JWH-018, M1 or an equivolume of vehicle. Mice were then placed into a new cage with fresh bedding to stimulate exploratory behavior. Temperature and locomotor activity data were collected at regular 5-min intervals and processed simultaneously by the Vital View data acquisition system (Mini Mitter Co., Inc.) for at least 8 hrs.

### Statistical Analysis

Curve fitting and statistical analyses for *in vitro* experiments were performed using GraphPad Prism version 4.0b (GraphPad Software Inc., San Diego, CA). The Cheng-Prusoff equation [61] was used to convert the experimental IC_50_ values obtained from competition receptor binding experiments to K_i_ values, a quantitative measure of receptor affinity. Non-linear regression for one-site competition was used to determine the IC_50_ for competition receptor binding. Curve fitting of concentration-effect curves via non-linear regression was also employed to determine the EC_50_ (a measure of potency) and E_max_ (a measure of efficacy) for [^35^S]GTPγS experiments. Data are expressed as mean ± SEM. A one-way ANOVA, followed by Tukey's Multiple Comparison *post-hoc* Test, was used to determine statistical significance (*P*<0.05) between three or more groups.

For core body temperature experiments, the area under the curve (AUC) was calculated using a trapezoidal rule from 0–10 hr. For locomotor activity, total locomotor counts were summed from 0–10 hr. For temperature data, statistical significance (*P*<0.05) was determined using a one-way ANOVA, followed by Tukey's HSD *post-hoc* test. Locomotor data were not normally distributed; therefore, a Kruskal-Wallis one-way ANOVA on ranks was performed, and all pairwise comparisons were then made using the Tukey's HSD test. All *in vivo* statistical calculations were performed using SigmaStat 3 (Systat Software, Inc., San Jose, CA).
